# KITENIN promotes glioma invasiveness and progression, associated with the induction of EMT and stemness markers

**DOI:** 10.18632/oncotarget.3087

**Published:** 2014-12-26

**Authors:** Kyung-Hwa Lee, Eun-Jung Ahn, Se-Jeong Oh, Ok Kim, Young-Eun Joo, Jeong-A Bae, Somy Yoon, Hyang-Hwa Ryu, Shin Jung, Kyung-Keun Kim, Jae-Hyuk Lee, Kyung-Sub Moon

**Affiliations:** ^1^ Department of Pathology, Chonnam National University Hwasun Hospital and Medical School, Hwasun, Jeollanam-do, South Korea; ^2^ Department of Neurosurgery, Chonnam National University Hwasun Hospital and Medical School, Hwasun, Jeollanam-do, South Korea; ^3^ Department of Internal Medicine, Chonnam National University Hwasun Hospital and Medical School, Hwasun, Jeollanam-do, South Korea; ^4^ Medical Research Center of Gene Regulation and Center for Creative Biomedical Scientists, Chonnam National University Medical School, Gwangju, South Korea

**Keywords:** Neoplastic stem cell, Epithelial-mesenchymal transition, Glioma, Neoplasm invasiveness, Human VANGL1 protein

## Abstract

KITENIN (KAI1 COOH-terminal interacting tetraspanin) promotes tumor invasion and metastasis in various cancers. This study assessed the association between KITENIN expression and advanced glioma grade in patients. In vitro assays revealed that KITENIN knockdown inhibited the invasion and migration of glioma cells, whereas KITENIN overexpression promoted their invasion and migration. In orthotopic mouse tumor models, mice transplanted with KITENIN-transfected glioma cells had significantly shorter survival than mice transplanted with mock-transfected cells. Patients with low KITENIN expression showed a significantly longer progression-free survival than patients with high KITENIN expression. KITENIN induced the expression of the epithelial-mesenchymal transition (EMT) markers (N-cadherin, ZEB1, ZEB2, SNAIL and SLUG) as well as the glioma stemness markers (CD133, ALDH1 and EPH-B1). Taken together, these findings showed that high levels of KITENIN increased glioma invasiveness and progression, associated with the up-regulation of EMT and stemness markers.

## INTRODUCTION

Malignant gliomas are highly infiltrative tumors, with cells migrating from the primary lesion into surrounding normal brain tissue. The invasive phenotype is key to the clinical progression of malignant glioma, complicating complete surgical resection and permitting tumor regrowth and further invasion of surviving tumor cells. In addition, glioblastoma multiforme (GBM), the most malignant type of glioma (World Health Organization (WHO) grade IV), has been regarded as a representative human cancer with natural intratumoral heterogeneity [1]. Due to the presence in these tumors of diverse cell populations with different biological properties, especially cells resistant to chemotherapy or radiotherapy, malignant gliomas show aggressive recurrence in clinical scenario [2]. According to the cancer stem cell theory, glioma stem cells are a small population of tumor cells that initiate and propagate tumors [3]. Moreover, glioma stem cells are more invasive and intractable to therapeutic modalities in tumor microenvironments [4, 5]. Invasiveness and glioma stemness are a lethal combination, resulting in the progression of malignant gliomas and the failure of therapeutic modalities. The median survival of GBM patients has been reported to be about 15 months, despite standard treatment with concurrent radiochemotherapy and adjuvant chemotherapy with temozolomide [6].

Numerous molecular markers and pathways associated with the invasiveness of malignant gliomas have been explored. An essential feature of the invasiveness and metastasis of systemic cancers is the epithelial-mesenchymal transition (EMT), in which the expression of epithelial E-cadherin is reduced while the expression of mesenchymal cadherins such as N-cadherin and cadherin-11 is increased [7]. The EMT can induce cancer cells to acquire stemness within the cancer microenvironment, resulting in drug resistance [8]. However, the EMT has not been fully accepted as a key pathway of malignant glioma invasiveness, largely due to the presence of different cell types in these tumors. E-cadherin expression has been found to be low or absent in gliomas and normal brain tissue, whereas N-cadherin is extensively expressed [9, 10]. However, cadherin subtypes have also been reported to be expressed in various grades of glioma [9-12]. Therefore, it has not been determined whether classic E-to-N-cadherin conversion is essential for glioma invasion or progression.

KAI1/CD82, a 40–75-kDa transmembrane glycoprotein of the tetraspanin family, was initially identified as a suppressor of prostate-specific metastasis [13]. Down-regulation or loss of KAI1/CD82 has been associated with metastatic progression and poor prognosis in many human cancer types [14-16]. A spliced variant of KAI1 at the COOH-terminal region, however, showed a reduction in metastasis suppressor function, suggesting that this region is important for the effects of KAI1 on cell motility [17]. Subsequently, KAI1 COOH-terminal interacting tetraspanin (KITENIN), a binding partner of the KAI1/CD82 and a homolog of *Drosophila* VANGL1, was found to be specifically associated with the COOH-terminal region of KAI1/CD82 [18].

The function of KITENIN in carcinogenesis has not been fully elucidated. KITENIN overexpression was found to increase the tumorigenicity, invasiveness, and adhesion to fibronectine in of mouse colon cells [19]. KITENIN was also shown to function as a metastasis-enhancing gene [20] that participates in the dissemination of colorectal [21] and squamous cancer cells [22]. The interaction of KITENIN with Dishevelled (Dvl)/PKCδ was found to play an important role in regulating colorectal cancer cell invasion via extracellular signal-regulated kinase (ERK)/activating protein-1 (AP-1) activation [21]. KITENIN has also been associated with EGFR-independent EGF signals via the downstream pathway of the KITENIN/ErbB4-Dvl2-c-Jun axis [23]. In addition, small interfering RNA (siRNA) and microRNA-124 targeting KITENIN inhibited tumor metastasis in a mouse colon cancer model [19, 20, 24]. Assays of human tumor specimens showed that KITENIN expression was significantly higher in advanced-stage colon cancer and metastatic foci than in early-stage colon cancer [21].

The expression and biological role of KITENIN in malignant gliomas have not yet been determined, nor has the possible relationships of KITENIN expression with EMT and cancer stem cells. Previous findings on the activities of KITENIN in the invasiveness and metastatic potential of diverse cancers suggested that KITENIN may play a significant role in malignant gliomas. This study was therefore designed to assess the biological role of KITENIN *in vitro* and *in vivo* using orthotopic mouse glioma models, as well as to analyze the association between KITENIN expression and clinicopathological findings in patients with glioma, including their survival rates. In addition, the effects of genetic modulation of KITENIN on EMT and cancer stemness factors in malignant gliomas were analyzed.

## RESULTS

### KITENIN expression is increased in human glioma tissues

**Figure 1:**
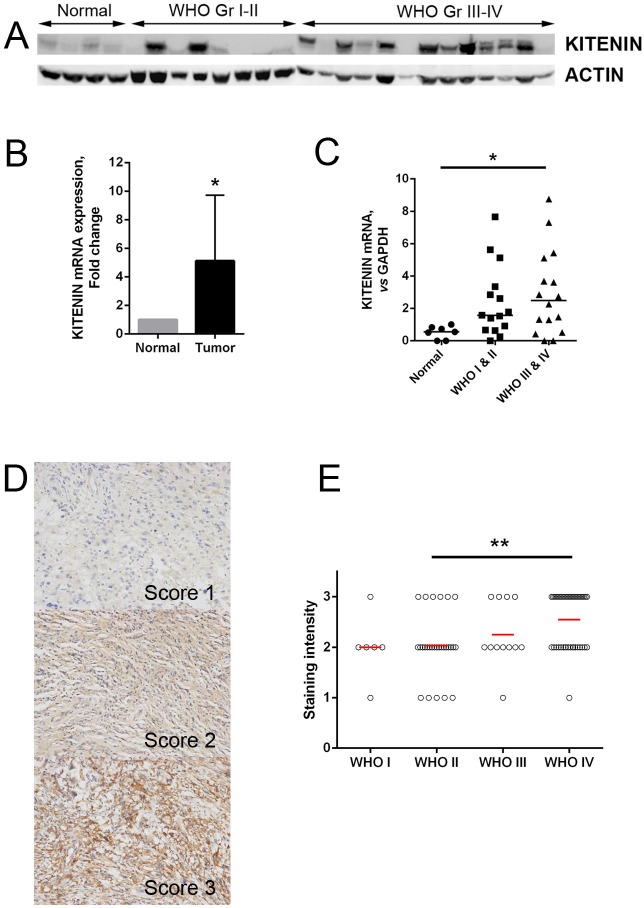
KITENIN expression in human glioma samples (A) Western blotting analysis showing that the overall expression of KITENIN tended to increase along the sequence from normal brain tissues to low-grade gliomas (WHO grades I-II), and then to high-grade gliomas (WHO grades III-IV). (B) qRT-PCR showing that the levels of expression of KITENIN mRNA were significantly higher in glioma tissues than in normal brain tissues (*P*=0.010). (C) Relative KITENIN mRNA levels, normalized to GAPDH mRNA levels, were significantly higher in tumor than in normal tissue (*P*=0.037). (D) Immunohistochemistry with anti-KITENIN antibody on human glioma tissue samples showing cytoplasmic positivity, with representative figures according to staining intensity. (E) KITENIN expression, represented by the staining intensity from WHO tumor grade I through to grade IV, showing statistically significant differences between tumor grade groups (*P*=0.001, Table 5). The bar graphs show the mean ± the standard error of the mean (SEM) for each group. (*P<0.05, **P<0.01).

The expression of KITENIN in frozen glioma and normal brain tissues was evaluated at both the mRNA and protein levels. Compared with normal brain tissues, KITENIN protein expression was significantly higher in glioma tissues, with a greater increase in high-grade (WHO grades III and IV) than low-grade (WHO grades I and II) gliomas (Fig. 1A). The levels of expression of KITENIN mRNA were also significantly higher in glioma than in normal brain tissues (Fig. 1B) and were significantly higher in high-grade than low-grade gliomas (Fig. 1C).

Immunohistochemical assessments of KITENIN expression in glioma tissue samples from 86 patients showed that this protein was highly expressed in seven of 33 low-grade gliomas (21.2%) and in 28 of 53 high-grade tumors (52.8%) (Fig. 1D). The difference between these groups was statistically significant (*P*=0.004, Table 1), with high KITENIN expression correlating with the increase in tumor grade (*P*=0.001, Fig. 1E). KITENIN expression was not significantly correlated with patient age, sex, tumor size, tumor location, severity of edema, or presence of cystic change (*P*>0.05 each, Table 1).

**Table 1:** Correlations of KITENIN expression with preoperative clinicopathological features in glioma patients

Variables		No. of cases	KITENIN expression		*p* Value
			Low	High	
Age	< 60 years	60	39	21	0.102
	≥ 60 years	26	12	14	
Sex	Male	41	21	20	0.145
	Female	45	30	15	
Tumor size	< 4.5cm	46	28	18	0.751
	≥ 4.5cm	40	23	17	
Location	Non-eloquent	43	24	19	0.510
	Eloquent	43	27	16	
Edema	None to mild	40	24	16	0.902
	Moderate to severe	46	27	19	
Cystic change	Absent	37	21	16	0.676
	Present	49	30	19	
WHO grade	1	6	26	7	0.004
	2	27			
	3	10	25	28	
	4	43			

### Modulation of KITENIN expression affects the invasiveness and migration of glioma cell lines

**Figure 2:**
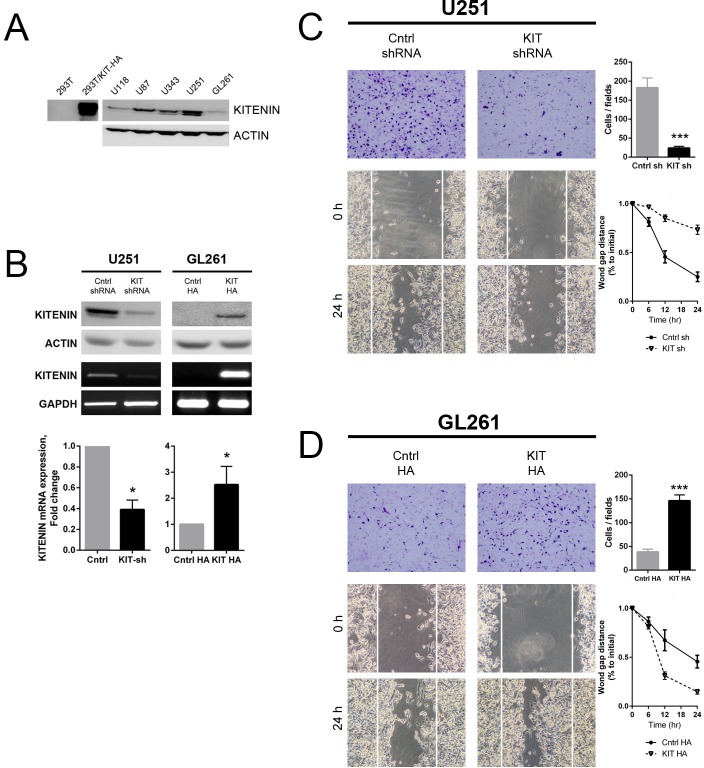
Effect of KITENIN overexpression and knockdown on the invasion and migration of human and mouse glioma cell lines (A) Western blotting analysis showing baseline expression of KITENIN in various glioma cell lines and 293T cells. (B) Western blotting, RT-PCR, and qRT-PCR analysis of KITENIN protein and mRNA expression in the U251 and GL261 cell lines at baseline, after stable knockdown in U251 cells and after stable overexpression in GL261 cells. (C) KITENIN knockdown significantly decreased U251 cell invasion and migration, as shown by cell counts per field (*P*<0.001) and wound gap distance, respectively. (D) KITENIN overexpression significantly increased GL261 cell invasion and migration, as shown by cell counts per field (*P*<0.001) and wound gap distance, respectively. Bar graphs show the mean ± the standard error of the mean (SEM). (*P<0.05, ***P<0.001).

The functional role of KITENIN in gliomas was first assessed by evaluating the expression of KITENIN in various glioma cell lines. KITENIN was highly expressed in the U87, U343, and U251 cell lines, but only slightly expressed in U118 and GL261 cells (Fig. 2A). Accordingly, U251 cells were used for KITENIN knockdown, whereas GL261 cells were used for overexpression. Treatment of U251 cells with shKITENIN reduced KITENIN mRNA and protein expression, whereas transfection of KITENIN cDNA into GL261 cells increased the expression of both (Fig. 2B). KITENIN knockdown in U251 cells significantly reduced *in vitro* cell invasion (Fig. 2C, upper), whereas significantly greater number of GL261 cells with stable KITENIN overexpression (KIT HA) migrated through the membrane compared with mock-transfected (cntrl HA) GL261 cells (Fig. 2D, upper). After 24 hours, the artificial wound gaps became significantly narrower in plates of mock-transfected (cntrl shRNA) than sh-KITENIN-transfected (KIT shRNA) U251 cells (Fig. 2C, lower). The wound gaps in plates of mock-transfected GL261 cells remained wide open. The gaps were almost completly filled in plates of KITENIN-overexpressed GL261 cells (Fig. 2D, lower). Similar results were observed in other human glioma cell lines in which KITENIN expression was similarly modulated (Supplementary Fig. 1). KITENIN overexpression in U118 cells led an increase of invasiveness, whereas treatment of U343 cells with siKITENIN reduced cell migration through the membrane when compared with vector-transfected U343 cells.

### Modulation of KITENIN expression is associated with changes in EMT markers and regulators

**Figure 3:**
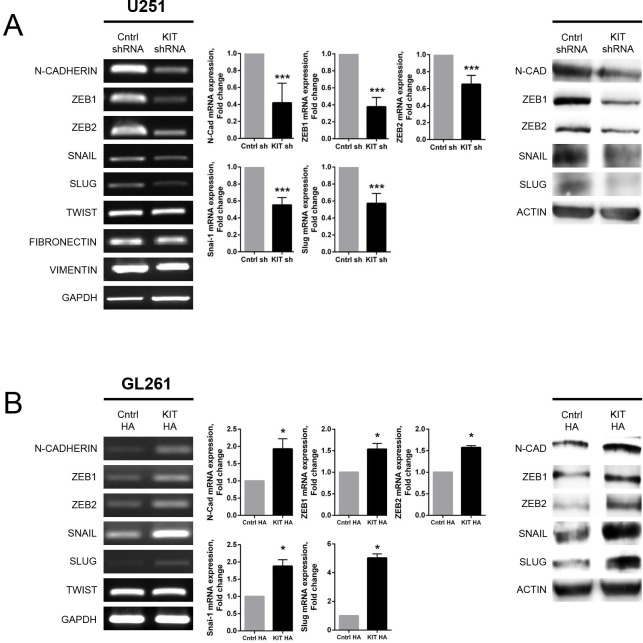
Altered expression of EMT markers associated with KITENIN modulation in U251 and GL261 cells (A) Stable knockdown of KITENIN in U251 cells decreased the levels of expression of N-cadherin, ZEB1, ZEB2, SNAIL, and SLUG mRNAs, as shown by RT-PCR (left panel) and qRT-PCR (middle panel), as well as protein levels, as shown by Western blotting (right panel). (B) Stable overexpression of KITENIN in GL261 cells increased the expression of EMT markers at the mRNA [RT-PCR (left panel), qRT-PCR (middle panel)] and protein [Western blot (right panel)] levels. Bar graphs show the mean ± the standard error of the mean (SEM). (*P<0.05, ***P<0.001).

To search for factors associated with alterations invasiveness and migration caused by modulation of KITENIN expression, the levels of expression of EMT markers and regulators were determined. Stable KITENIN knockdown in U251 cells reduced the expression of N-cadherin (*CDH2*), ZEB1, ZEB2, SNAIL (*SNAI1*), and SLUG (*SNAI2*) mRNA and protein (Fig. 3A). By contrast, stable overexpression of KITENIN in GL261 cells increased of N-cadherin, ZEB1, ZEB2, SNAIL, and SLUG mRNA and protein (Fig. 3B). Similar results were observed in KITENIN-overexpressed U118 cells (Supplementary Fig. 2A).

### Modulation of KITENIN expression affects stem cell marker expression and neurosphere- and colony-forming capacities

**Figure 4:**
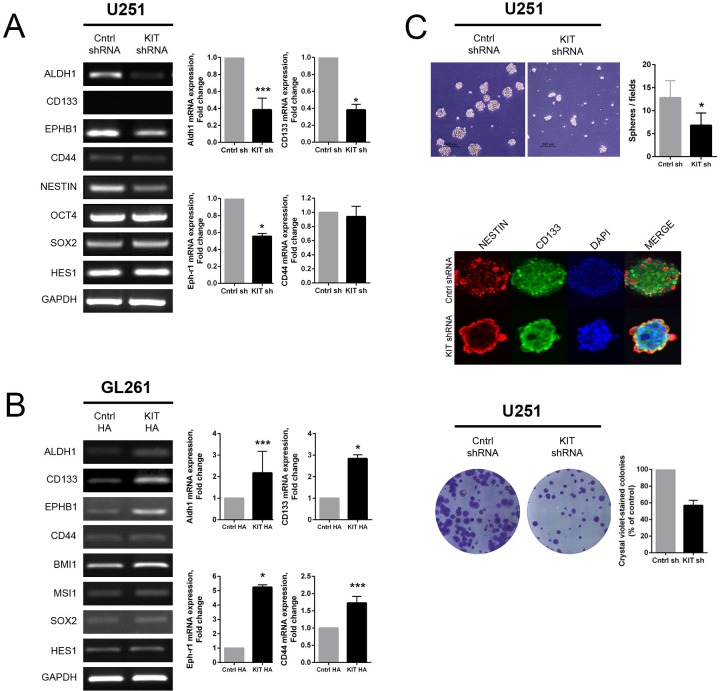
Effect of KITENIN overexpression and knockdown on features of glioma stemness-associated markers and functions (A) Stable knockdown of KITENIN in U251 cells decreased the expression of ALDH1, CD133, and EPH-B1 mRNAs, as shown by RT-PCR (left panel) and qRT-PCR (right panel). (B) Stable overexpression of KITENIN in GL261 cells increased the expression of mRNAs encoding glioma stemness markers, including ALDH1, CD133, EPH-B1, and CD44, as shown by RT-PCR (left panel) and qRT-PCR (right panel). (C) Stable KITENIN knockdown in U251 cells significantly reduced the number of neurospheres (upper panel), Nestin and CD133 expression centered on the peripheral rim of the neurospheres (middle panel), and colony-forming ability (lower panel). Bar graphs show the mean ± the standard error of the mean (SEM). (*P<0.05, ***P<0.001).

To investigate the possible role of KITENIN in cancer stemness, the relationships between CD133-positive cells and levels of KITENIN expression were evaluated in various glioma cell lines. The U343, U251 and U87 cell lines, with relatively high expression of endogenous KITENIN, showed a higher proportion of CD133 positivity than the U118 and GL261 cells lines, with low endogenous KITENIN levels (Supplementary Fig. 3). Stable KITENIN knockdown in U251 cells decreased the levels of ALDH1, CD133, and EPH-B1 mRNAs (Fig. 4A), whereas KITENIN overexpression in GL261 cells increased the levels of ALDH1, CD133, EPH-B1, and CD44 mRNAs (Fig. 4B). The expression of several stem cell markers was also increased in KITENIN-overexpressed U118 cells (Supplementary Fig. 2B). The number of neurospheres on day 7 was significantly lower in shKITENIN-treated than mock-transfected U251 cells (Fig. 4C, upper). The expression of representative glioma stem cell markers, Nestin and CD133, as determined using double immunofluorescent staining, was relatively even throughout the neurospheres cultured from mock-transfected U251 cells. In shKITENIN-treated U251 cells, however, the expression of Nestin and CD133 was centered on the peripheral rim of the neurospheres (Fig. 4C, middle). Moreover, shKITENIN-treated U251 cells had lower colony-forming ability than mock-transfected U251 cells (Fig. 4C, lower). In contrast, neurosphere- and colony-forming abilities were enhanced in KITENIN-overexpressed GL261 cells (Supplementary Fig. 4).

### Elevated expression of KITENIN is associated with shorter survival and increased invasiveness in orthotopic mouse glioma models

**Figure 5:**
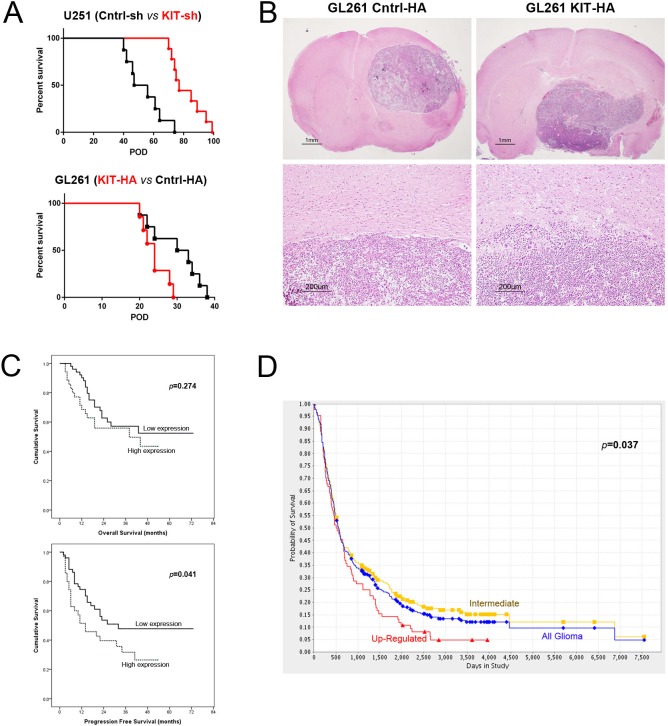
Kaplan–Meier survival analysis according to KITENIN expression level in orthotopic mouse tumor models and in cohorts of glioma patients (A) Mice transplanted with shKITENIN-treated U251 cells showed a longer median overall survival (OS) than mice transplanted with mock-treated U251 cells (71.0 *vs.* 49.0 days, *P*<0.001, upper graph), whereas mice transplanted with KITENIN-transfected GL261 cells had a shorter median OS than mice with mock-transfected GL261 cells (24 *vs.* 31.5 days, *P*=0.03, lower graph). (B) Histopathological examination of mouse brains showing that tumors in brains transplanted with KITENIN-transfected GL261 cells had more invasive and multi-lobulated features (right top and bottom) than tumors in brains with mock-transfected GL261 cells (left top and bottom). (C) Kaplan–Meier survival analysis according to KITENIN expression levels in a cohort of 86 glioma patients showing that median OS was shorter (32.8 *vs.* 47.5 months, *P*=0.274, upper graph) and median PFA was significantly shorter (*P*=0.041, lower graph) in patients with high than low KITENIN expression. (D) Kaplan–Meier survival analysis according to KITENIN expression levels in a cohort of 343 glioma patients in the REMBRANDT database of the National Cancer Institute showing that OS was significantly shorter in patients with high than intermediate KITENIN expression (*P*=0.037).

In orthotopic intracranial mouse tumor models, modulation of KITENIN expression was associated with altered invasiveness and survival rates. Median survival was significantly longer in mice transplanted with shKITENIN-treated than mock-treated U251 cells (71.0 *vs*. 49.0 days, *P*<0.001, Fig 5A, upper), but was significantly shorter in mice transplanted with KITENIN-transfected than mock-transfected GL261 cells (24 *vs*. 31.5 days, *P*=0.03, Fig 5A, lower). Histopathological examination showed smooth expansile tumor growth margins within the brains of mice with mock-transfected GL261 cells, but irregular tumor growth margins with infiltrative tumor cells within the brains of mice transplanted with KITENIN-transfected GL261 cells (Fig. 5B).

### Up-regulated KITENIN expression in glioma predicts a poor prognosis

In our glioma cohort, the median follow-up period after surgery was 44.9 months (range, 3 to 73 months). Although median overall survival (OS) was shorter in patients with high than low KITENIN expression, the difference was not statistically significant (32.8 *vs*. 47.5 months, *P*=0.274, Fig. 5C, upper). Univariate analyses showed that patient age and WHO tumor grades were significantly prognostic of OS (*P*<0.001 each; Supplementary Table 4). Moreover, multivariate Cox regression analyses showed that patient age (*P*=0.001) and WHO grade (*P*=0.005) were independently prognostic factors of OS, whereas KITENIN expression was not. By contrast, median progression-free survival (PFS) was significantly longer in patients with low than high KITENIN expression (42.0 *vs.* 23.8 months, *P*=0.041, Fig. 5C, lower). Univariate analysis showed that both patient age and WHO grade were significantly prognostic of PFS (*P*<0.001 each, Supplementary Table 5). Similarly, multivariate Cox regression analyses showed that patient age and WHO grade were independently prognostic of PFS (*P*<0.001 each).

Assessment of a cohort of 343 glioma patients provided by the REpository of Molecular BRAin Neoplasia DaTa (REMBRANDT) of the National Cancer Institute (https://caintegrator.nci.nih.gov/rembrandt/home.do) showed that levels of KITENIN expression were significantly correlated with OS. KITENIN was highly and intermediately expressed in 84 and 259 patients, respectively, with OS being shorter in the former group *(P*=0.037, Fig. 5D). Overall, these findings indicate that high KITENIN expression in glioma is predictive of poor patient prognosis.

## DISCUSSION

The findings shown here indicate that the invasiveness and migration of glioma cells were associated with their overexpression of KITENIN. Furthermore, genetic modulation of KITENIN altered the expression of EMT-related factors, including N-cadherin, ZEB1, ZEB2, SNAIL, and SLUG. Although the role of EMT in malignant gliomas remains unclear, several factors related to EMT or its reminiscent program (glial-mesenchymal transition) have been revealed as key molecules in glioma invasion and progression [25-31]. The strong correlation between KITENIN and EMT factors may therefore explain the enhanced invasiveness and migration of glioma cells. In addition, genetic modulation of KITENIN altered the expression of several factors related to glioma stemness, including CD133, CD44, ALDH1, and EPH-B1. Reduced KITENIN expression resulted in decreased neurosphere- and colony-forming abilities. These findings suggested that altered KITENIN expression is linked to changes in glioma stemness. In orthotopic glioma models, mice showed significantly increased survival following KITENIN knockdown but shorter survival with KITENIN overexpression. In contrast to smooth expansile tumor growth margins in the control group, mouse brains implanted with KITENIN-overexpressed cells showed irregular and infiltrative tumor growth margins. KITENIN expression was found to be higher in tumor samples from patients with more invasive and malignant gliomas (WHO grades III and IV) than in samples from patients with lower grade gliomas (WHO grades I and II) and normal tissue, similar to findings in other systemic cancers [20, 32, 33]. In agreement with the altered survival of mice transplanted with KITENIN-transfected cells, the survival data from human glioma patients showed that the up-regulation of KITENIN expression was closely associated with significantly shorter OS (REMBRANDT data) and PFS (out cohort). In comparison, expression of KITENIN did not correlate with patient age, sex, tumor size or location, severity of edema, or presence of cystic changes.

Invasion of malignant glioma through the white matter track has been found to lead to tumor recurrence at a more inaccessible or deeper site than the initial location, resulting in a dismal prognosis. Cancer stem cells have been regarded as a small population, responsible for the initiation of tumor burden and therapeutic refractoriness. Apart from the presence of conventional EMT in malignant gliomas, genome-based large cohort studies using GBM specimens and analyzing clinical outcomes defined a mesenchymal subgroup of GBM [34, 35], characterized by shorter PFS and OS due to aggressive dissemination and resistance to chemoradiotherapy [25, 34, 35]. Moreover, the reminiscent program of EMT (i.e., glial-mesenchymal transition) was found to enhance glioma progression and invasion [25-31], chemoresistance [36], and biological changes after radiation [37]. Enrichment of the EMT pathway was also observed in radioresistant phenotypes [38] and was found to be greater in GBMs than in anaplastic astrocytomas [39], suggesting that EMT may be predictive of prognosis in glioma patients. Several investigations have also shown a link between EMT and glioma stemness [37, 40-43]. Subsequent to the finding that glioma stemness was co-regulated by Twist1 and Sox2 [42], normal neural stem cells that acquired glioma-like invasiveness through the combined FGF2 and BMP4 pathways were found to demonstrate EMT-related features [41]. ZEB1, an EMT inducer, was recently shown to be key in linking glioma stemness, invasiveness, and the expression of the chemoresistant enzyme *O*-6-methylguanine DNA methyltransferase (MGMT) [43]. In addition, the SNAIL (*SNAI1*) gene was found to enhance irradiation-induced glioma progression via EMT and glioma stemness [37].

The invasiveness and metastasis functions of KITENIN were shown to be mediated by ERK/AP-1 activation through the interaction of KITENIN with Dvl/PKCδ [21] or the KITENIN/ErbB4-Dvl2-c-Jun axis, as an EGFR-independent EGF signal [23] in colon cancer cells. As EMT is one of the pathways mediated by mitogen-activated protein kinase (MAPK) signaling [44, 45], KITENIN may be linked to EMT through the MAPK signaling pathway. Indeed, this link was explored in our preliminary experiment, in which the expression of KITENIN and EMT markers was assayed in human glioma samples (Supplementary Fig. 5). However, it is difficult to connect the downstream axis of KITENIN with enhanced cancer stemness. The JNK-c-Jun/AP-1 signaling pathway was shown to play a key role in mediating the induction of apoptosis in glioma stem cells [46]. However, JNK signaling has also been reported to be a critical pathway for glioma stemness [47] beyond gliomagenesis [48] and to correlate with histological grade [49]. Glioma stem cells from mesenchymal GBM were recently reported to show more aggressive features *in vitro* and in a xenograft mouse model [50]. Moreover, mesenchymal markers such as CD44 and vimentin were found to be maintained by ALDH1A3 [50]. Several EMT markers or inducers, including Twist1 [42], ZEB1 [43], and SNAIL [37], have been reported to enhance glioma stemness, leading to cancer progression and therapeutic resistance. Although this study did not show a direct connection between KITENIN expression and the EMT pathway, previous results have indicated that the JNK-c-Jun signaling pathway, the pivotal downstream axis of KITENIN, may be responsible for the enhancement of EMT and cancer stemness or the linkage between the two major obstacles in malignant gliomas.

In conclusion, we found that KITENIN was involved in glioma invasion and progression possibly through the induction of EMT and cancer stemness. Clinical findings showing that KITENIN expression was higher in high-grade gliomas associated with poor clinical outcome suggest that KITENIN may be a potential diagnostic and therapeutic target for the treatment of malignant gliomas. Although this study did not completely resolve the complicated connections of KITENIN with EMT and cancer stemness, KITENIN was shown to be correlated with glioma invasiveness. Better understanding of the molecular mechanisms that sustain the invasiveness of malignant glioma may indicate more effective therapeutic strategies. Further studies are needed to investigate the mechanistic links between KITENIN and EMT and/or cancer stemness in malignant gliomas.

## METHODS

### Glioma human tissue specimens and clinical data

Glioma specimens were obtained from 86 patients who had undergone operations at Chonnam National University Hwasun Hospital between 2007 and 2012. Diagnostic criteria for glioma grading were based on the WHO classification [1]. Thirty-one fresh frozen glioma tissue specimens, including 15 low-grade (WHO grades I and II) and 16 high-grade (WHO grades III and IV) gliomas, were obtained from patients within 30 min after resection. Samples were snap frozen in liquid nitrogen and stored at −80°C until use. Seven normal brain tissue specimens were obtained from trauma or cerebral infarction patients who underwent partial resection of normal brain as decompression treatment for severe head injuries. These patients or their legal surrogates provided written informed consents for the surgical procedures, as well as for the use of resected tissue specimens. Data were collected from medical records. Clinicopathologic characteristics, including tumor size and location, severity of perilesional edema, and presence of cystic changes, were based on preoperative magnetic resonance imaging (MRI). OS was calculated as the time from the date of surgery to the date of death or the last follow-up visit. PFS was also calculated as the time from the date of surgery to the date of recurrence, progression, or death. This study was approved by the institutional review board of the Chonnam National University Hwasun Hospital (CNUHH-2014-030).

### Tissue microarray construction and immunohistochemistry

Hematoxylin and eosin stained sections of gliomas were reviewed, and areas with a high density of glioma cells were selected for sampling in tissue microarrays, as described [51]. Areas of high-grade gliomas containing necrotic changes were excluded as much as possible. Three cores of each specimen were transferred to the donor blocks. The arrays were constructed with a 2 mm punch on a Beecher arrayer (Beecher Instruments, Sun Prairie, WI, USA). Seven tissue array blocks were prepared. A series of 4 mm sections were consecutively cut with a microtome and transferred to adhesive-coated slides. One section from each tissue array block was stained with hematoxylin and eosin. Slides were reviewed to determine whether the sample was representative of the original tumor.

Tissue microarray sections were immunostained with specific antibodies against KITENIN (1:200, Atlas, Stockholm, Sweden) using a Bond-max system (Leica Microsystems, Bannockburn, IL, USA). Programmed heat-induced epitope retrieval was carried out using bond epitope retrieval solution 1 (containing citrate buffer at pH 6.0). Negative controls were processed similarly in the absence of primary antibodies. Staining results were interpreted by two pathologists (LJH and LKH) who were blinded to the clinical findings. KITENIN stained the cytoplasm of glioma cells. The staining intensity of cancer cells was initially graded according to the following criteria: 0, no staining; 1, weak staining; 2, moderate staining; and 3, strong staining. However, as no glioma sample was immunonegative for KITENIN, samples were grouped into those with staining intensities of 1-2 (low expression) and 3 (high expression).

### Cell culture and transfection

Human glioma cells (U118, U87, U343, and U251) and human embryonic kidney (293T) cells were obtained from the American Type Culture Collection (ATCC; Manassas, VA, USA). The mouse glioma cell line, GL261, was kindly provided by Dr. Maciej S. Lesniak (University of Chicago). Cells were cultured in Dulbecco's modified Eagle's medium (DMEM; Hyclone^TM^, Thermo Scientific, Waltham, MA, USA) supplemented with 10% fetal bovine serum (FBS, GIBCO^®^, Invitrogen, Carlsbad, CA, USA) in a humidified atmosphere of 5% CO_2_ at 37°C. For KITENIN knockdown, pSUPER vectors (OligoEngine, Seattle, WA, USA) containing siRNA (5′-GCUUGGACUUCAGCCUCGUAGUCAA-3′) were constructed and transfected into U251 and U343 cells (KIT shRNA) using Lipofe amine^TM^ 2000 (Invitrogen) according to the manufacturer's instructions [21, 23]. Empty pSUPER vectors without siRNA were used as negative controls (Cntrl shRNA). KITENIN knockdown was confirmed at the mRNA and protein levels. Expression constructs of hemagglutinin (HA)-tagged mouse/human KITENIN were generated and transfected into GL261 and U118 cells using FuGene 6 (Roche, Indianapolis, IN, USA) (KIT HA), as described [19]. Empty hemagglutinin (HA) vectors without KITENIN tagging were used as negative controls (Cntrl HA). Clones of GL261 cells were selected in the presence of antibiotics. KITENIN overexpression in surviving clones was confirmed at the mRNA and protein levels.

### Reverse transcription polymerase chain reaction (RT-PCR) and quantitative real-time PCR (qRT-PCR)

Total RNA from cells and tumor tissues was extracted using Trizol reagent (Invitrogen). An adequate amount of RNA was reverse transcribed using LeGene Express 1^st^ Strand cDNA Synthesis System Kit (LeGene Biosciences, San Diego, CA, USA). Primer sequences used in this study are listed in Supplementary Tables 1 and 2. RT-PCR and qRT-PCR were performed as described [19, 21]. PCR amplification of cDNA was performed using gene-specific primers and h-Taq DNA Polymerase (SolGent, Daejeon, South Korea) under the following conditions: 15 min of denaturation at 95°C, followed by 35 cycles of denaturation for 30 sec at 95°C, annealing for 30 sec at 60°C, and extension for 30 sec at 72°C, followed by a final extension for 7 min at 72°C. PCR cycles were limited to 35. Primers for endogenous reference gene (GAPDH) were used as internal controls. PCR products were analyzed by electrophoresis on agarose gels containing ethidium bromide.

qRT-PCR analysis was performed using a VeriQuest SYBR Green PCR kit (Affymetrix, Santa Clara, CA, USA) and a CFX96 Touch^TM^ Real-time PCR Detection System (Bio-rad Laboratories) with CFX manager software (Bio-rad Laboratories, version 3.1.1517.0823). The following amplification parameters were used: 10 min hot start at 95°C, followed by 40 cycles of 15 sec of denaturation at 95°C, 30 sec of annealing at 60°C, and 30 sec of elongation at 72°C. Target gene expression was normalized relative to the expression of GAPDH in the same sample. All reactions were performed in triplicate, with the experiments independently repeated at least three times and the average calculated. Relative quantification was calculated using the 2^−ΔΔ^ method.

### Western blot analysis

Cells and tissues were lysed by incubation in a buffer (50 mM Tris–HCl, pH 7.4, containing 150 mM NaCl, 1% Nonidet P-40, 0.1% SDS, 0.1% deoxycholic acid, 10 mM NaF, 10 mM Na_4_P_2_O_7_, 0.4 mM Na_3_VO_4_, and protease inhibitors) for 30 min on ice. After centrifugation, proteins (30 μg) were separated by 10% polyacrylamide gel electrophoresis containing 0.1% SDS and electrophoretically transferred to nitrocellulose membranes. After blocking with 3% nonfat milk in phosphate buffered saline (PBS)-Tween 20 buffer at room temperature for 1 hour, the nitrocellulose membranes were sequentially incubated with specific primary antibodies and anti-rabbit or anti-mouse immunoglobulin secondary antibodies conjugated to horseradish peroxidase (HRP). The primary and secondary antibodies used in this study are listed in the Supplementary Table 3. Proteins were quantified using an electrochemiluminescence (ECL) system (Pierce Biotechnology, Rockford, IL USA).

### Cell invasion and migration assay

Cell invasion was measured using a Chemotaxis chamber (Neuro Probe, Gaithersburg, MD, USA). Gene-modulated human and mouse glioma cells (1×10^5^ U251, U343, and U118 cells and 5×10^4^ GL261 cells in 0.35 ml of serum-free DMEM) were loaded into the top chamber of a 10-well invasion chamber assay plate. Subsequently, complete DMEM (DMEM supplemented with 10% FBS) was placed in the lower chambers, and a matrigel-coated membrane was inserted between the two chambers. After incubation for 24 hours at 37°C, the membranes were fixed and stained with a hemacolor rapid staining kit (Merck, Darmstadt, Germany). Cells from five random microscopic fields (each 0.5 mm^2^) were counted using a hematocytometer under a light microscope. Results are reported as the mean ± standard error of the number of cells per field. Each experiment was repeated independently at least three times.

Gene-modulated U251 cells and GL261 cells in pairs were seeded into six-well plates at a density of 1×10^5^ cells/well and cultured to 90% confluence in DMEM supplemented with 10% FBS for 24 h. The cell culture media were removed from the wells and a straight transverse line through the adherent cells was drawn using a ruler and a sterile plastic 200 μl micropipette tip, resulting in a uniform gap. Serum-free DMEM was added, and the distances between the gaps were measured after 0, 6, 12, and 24 hours, following the capture of six random sites by the microscopic field of view, with the distances normalized to 100%.

### Neurosphere culture

Gene-modulated glioma cells were plated and grown in complete DMEM. Two days later, the cells were washed twice and seeded at a density of 1×10^5^ cells/ml in a serum-free Neurobasal Medium (21103-049, GIBCO) supplemented with B27 (12587-101, GIBCO), 50 ng/ml epidermal growth factor (EGF, BD Biosciences, San Jose, CA, USA), and 50 ng/ml fibroblast growth factor (FGF, BD Biosciences). Primary neurospheres were cultured for 7 days, followed by collection and passage by mechanical dissociation. Viable cells were counted by trypan blue exclusion. For secondary neurosphere assays, approximately 20,000 to 100,000 viable cells were resuspended in proliferation medium and plated at a density of 2×10^4^ cells/ml in each well of a 24-well plate (Corning Incoporated, Corning, NY, USA). Cells were allowed to form spheres for 10 days and the number of neurospheres was counted by microscopy.

### Colony forming assay

Gene-modulated glioma cells were added to six-well plates at a density of 120 cells per well and cultured for 14 days and fed two twice weekly with cell culture media. The cells were fixed with 100% methanol and stained with 5% crystal violet. Colonies of more than 50 cells were manually counted. Data reported represent the average of three experiments.

### Mouse orthotopic intracranial glioma model

BALB/c nude mice were injected with U251 cells and C57BL/6 mice with GL261 cells. Briefly, 6- to 8-week-old male mice weighting 15–18 g were purchased from OrientBio (Seongnam, South Korea). Mice were fed autoclaved pelleted food and water ad libitum. The experimental protocol was approved by the Chonnam National University Medical School Research Institutional Animal Care … Use Committee. Maintenance of animals and all *in vivo* experiments were performed according to the *Guiding Principles in the Care and Use of Animals* (DHEW publication, NIH 80-23).

For intracranial injection, mice were anesthetized with an intraperitoneal injection of ketamin hydrogen chloride solution. A burr hole 1 mm in diameter in the right hemisphere was made 2 mm lateral to the sagittal suture and 2 mm posterior to the coronal suture. Using stereotactic apparatus (KDS310, KD Scientific, Holliston, MA, USA), tumor cells (5 × 10^5^ cells of gene-modulated U251 and 2 × 10^5^ cells of gene-modulated GL261 cells in 2.5 μL of saline) were injected through a 26-gauge Hamilton syringe (Hamilton Company, Reno, NV, USA) at a depth of 3 mm over a 5-minute period. Each cell line was injected into eight mice per group. Tumor-bearing animals were checked daily for neurological symptoms and weight. Animals that showed symptoms such as apathy, severe hunchback posture, decreased motion or activity, messy fur, dragging legs, or drastic loss of body weight were euthanized by cervical dislocation. Mouse brain specimens were extracted immediately after euthanization and fixed in 10% neutral-buffered formalin for 3 days. Brains were dissected, embedded in paraffin, and stained with hematoxylin for histopathological evaluation.

### Immunofluorescence staining and flow cytometry

Cells were washed three times in PBS, fixed in 4% paraformaldehyde for 10 min at room temperature, and permeabilized with PBS containing 0.25% of Triton X-100 (PBST) for 10-15 min at room temperature. After three washes with PBS, cells were blocked with 1% bovine serum albumin (BSA) for 30 min. Samples were incubated overnight at 4 ºC with anti-Nestin (1:500, 611659, BD Transduction Laboratories) and anti-CD133 (1:100, LF-PA50121, AB Frontier, Seoul, South Korea) antibodies, followed by incubation with Alexa Flour 488 goat anti-mouse IgG or Alexa Flour 568 goat anti-rabbit IgG (Molecular Probes, Grand Island, NY, USA) for 1 hour at room temperature. After three washes with PBS, immune-labeled cells were counterstained with 50 μl of DAPI at 37 ºC for 10 min. Cells were examined with a Laser Scanning Confocal Microscope (LSM510, Zeiss, Oberkochen, Germany).

Flow cytometry analysis of CD133 expression was performed by suspending 2×10^5^ cells of each glioma cell line in cold PBS. Human cells were stained with phycoerythrin (PE)-conjugated anti-human CD133 (130-080-801, Miltenyi Biotec, Auburn, CA, USA) and isotype antibodies (130-092-212), whereas mouse cells were stained with allophycocyanin (APC)-conjugated anti-mouse CD133 (17-1331, eBioscience, San Diego, CA, USA) and isotype antibodies (17-4301) according to the manufacturer's instructions. Flow cytometry was performed using a FACSCalibur (BD Biosciences, San Jose, CA, USA) and Flowjo analysis software (TreeStar, Ashland, OR, USA).

### Patient datasets and data analysis from REMBRANDT

Data on glioma patients were publicly available in de-identified form from the NCI Repository for Molecular Brain Neoplasia Data (REMBRANDT) database (https://caintegrator.nci.nih.gov/rembrandt/login.do) [52]. KITENIN expression and overall survival were correlated in samples from 343 glioma patients through to May 13, 2014. Data were used to construct graphs for Affymetrix reporters 219330 at the Highest Geometric Mean Intensity and associated survival. Up-, down-, or intermediate expression of glioma specimens referred to ≥ 2-fold changes in KITENIN expression compared with specimens from nonglioma patients. Differences in survival were assessed by the log-rank test.

### Statistical analysis

Relationships between protein expression and categorical variables were compared using Chi-square tests or Fisher's exact probability tests, as appropriate. The effects of single variables on survival were determined by univariate and multivariate analyses. For multivariate analysis, independent prognostic factors were determined using Cox's proportional hazards model. Some variables that could be dependent on other variables were excluded from the model. Survival curves were calculated by the Kaplan-Meier method and compared by log-rank tests. GraphPad Prism version 6.00 software program for Windows (GraphPad, La Jolla, CA, USA) was used to analyze *in vitro* and *in vivo* experiments, with the data presented as the mean ± the standard error of the mean (SEM). All data were analyzed using SPSS version 20.0 software program for Windows (SPSS, Chicago, IL, USA). Statistical significance was defined as a *P* value < 0.05.

## SUPPLEMENTARY MATERIAL TABLES AND FIGURES


