# Supplementary material for: KITENIN promotes glioma invasiveness and progression, associated with the induction of EMT and stemness markers
Source: Oncotarget. 2014 Dec 26;6(5):3240–53. doi: 10.18632/oncotarget.3087 (PMC4413650; doi:10.18632/oncotarget.3087)
Supplement: Supplementary file 1 [file oncotarget-06-3240-s001.pdf]

KITENIN promotes glioma invasiveness and progression, associated with the induction of EMT and stemness markers

Supplementary Material

Supplementary Table 1: Human gene primers used for RT-PCR and qRT-PCR.

Gene	Primer type	Primer sequence	Product size
KITENIN	Forward	5'-CGG AAT AAA GAC GGC AGA GG-3'	152bp
	Reverse	5'-TGC TCC GAG GTG CCT GTG AT-3'	
CDH2	Forward	5'-TCA GGC GTC TGT AGA GGC TT-3'	94bp
	Reverse	5'-ATG CAC ATC CTT CGA TAA GAC TG-3'	
ZEB1	Forward	5'-ATG ACA CAG GAA AGG AAG G-3'	158bp
	Reverse	5'-AGC AGT GTC TTG TTG TAG-3'	
ZEB2	Forward	5'-CAA GAG GCG CAA ACA AGC C-3'	128bp
	Reverse	5'-GGT TGG CAA TAC CGT CAT CC-3'	
SNAI1	Forward	5'-TCG GAA GCC TAA CTA CAG CGA-3'	140bp
	Reverse	5'-AGA TGA GCA TTG GCA GCG AG-3'	
SLUG	Forward	5'-CGA ACT GGA CAC ACA TAC AGT G-3'	87bp
	Reverse	5'-CTG AGG ATC TCT GGT TGT GGT-3'	
TWIST	Forward	5'-GTC CGC AGT CTT ACG AGG AG-3'	156bp
	Reverse	5'-GCT TGA GGG TCT GAA TCT TGC T-3'	
Fibronectin	Forward	5'-CGG TGG CTG TCA GTC AAA G-3'	130bp
	Reverse	5'-AAA CCT CGG CTT CCT CCA TAA-3'	
Vimentin	Forward	5'-GAC GCC ATC AAC ACC GAG TT-3'	238bp
	Reverse	5'-CTT TGT CGT TGG TTA GCT GGT-3'	
Aldh1a3	Forward	5'-AAA TCC AGG GCA AGA CCA TC-3'	121bp
	Reverse	5'-TTC CAC ACC AGC ATC AGC AG-3'	
CD133	Forward	5'-GGA CCC ATT GGC ATT CTC-3'	171bp
	Reverse	5'-CAG GAC ACA GCA TAG AAT AAT C-3'	
EPH B1	Forward	5'-GAA GGT AAA CAC AGA AGT CAG G-3'	169bp
	Reverse	5'-CCC TGT CAT AGT CTC TGG AAA C-3'	
CD44	Forward	5'-TGA ATA TAA CCT GCC GCT TTG-3'	73bp
	Reverse	5'-TCC GTC CGA GAG ATG CTG TAG-3'	
Nestin	Forward	5'-GGC GCA CCT CAA GAT GTC C-3'	127bp
	Reverse	5'-CTT GGG GTC CTG AAA GCT G-3'	
OCT4	Forward	5'-GAC CAT CTG CCG CTT TGA GGC TCT G-3'	301bp
	Reverse	5'-GCG CCG GTT ACA GAA CCA CAC TCG G-3'	
SOX2	Forward	5'-GC TGG GCG CCG AGT GGA-3'	443bp
	Reverse	5'-GG CGA GC GTT CAT GTA GGT CTG-3'	
HES 1	Forward	5'-GCA CAG AAA GTC ATC AAA G-3'	162bp
	Reverse	5'-TGC TTC ACT GTC ATT TCC-3'	
GAPDH	Forward	5'-AGT TGT CAT GGA TGA CCT TGG C-3'	283bp
	Reverse	5'-ATC ACC ATC TTC CAG GAG CGA-3'	

Supplementary Table 2: Mouse gene primers used for RT-PCR and qRT-PCR.

Gene	Primer type	Primer sequence	Product size
KITENIN	Forward	5'-CAA GGA ATA AAG ACG GCA GAG G-3'	154bp
	Reverse	5'-TGC TCT GAG GTG CCT GTG AT-3'	
CDH2	Forward	5'-AGC GCA GTC TTA CCG AAG G-3'	101bp
	Reverse	5'-TCG CTG CTT TCA TAC TGA ACT TT-3'	
ZEB1	Forward	5'-TGG GAA AGC GTT CAA GTA CAA A-3'	254bp
	Reverse	5'-TTG GTT TAC AGA AAG CGG TTC TT-3'	
ZEB2	Forward	5'-AGC CAA GGA ATG CTA CCA A-3'	143bp
	Reverse	5'-GGC CCC AGA GCA TCA TAA TC-3'	
SNAI1	Forward	5'-GCC GGA AGC CCA ACT ATA GCG A-3'	199bp
	Reverse	5'-TTC AGA GCG CCC AGG CTG AGG TAC T-3'	
SLUG	Forward	5'-GCTCCACTCCACTCTCCTTT-3'	244bp
	Reverse	5'-CCAGCCCAGAGAACGTAGAA-3'	
TWIST	Forward	5'-CGG GTC ATG GCT AAC GTG-3'	197bp
	Reverse	5'-CAG CTT GCC ATC TTG GAG TC-3'	
Aldh1a3	Forward	5'-ATT TAT CAA CAA CGA CTG GCA C-3'	112bp
	Reverse	5'-CAC ATC GGG CTT ATC TCC TTC-3'	
CD133	Forward	5'-CTC ATG CTT GAG AGA TCA GGC-3'	222bp
	Reverse	5'-CGT TGA GGA AGA TGT GCA CC-3'	
EPH B1	Forward	5'-AGC TTT GGG CCT CTT ACT AG-3'	148bp
	Reverse	5'-CTC CTG TCA TGG TTT CTG G-3'	
CD44	Forward	5'-ATG GCC GCT ACA GTA TCT CC-3'	178bp
	Reverse	5'-GCA CAG ATA GCG TTG GGA TG-3'	
BMI1	Forward	5'-GAG CAG ATT GGA TCG GAA AG-3'	95bp
	Reverse	5'-GCA TCA CAG TCA TTG CTG CT-3'	
MSI1	Forward	5'-CGA GCT CGA CTC CAA AAC AAT-3'	304bp
	Reverse	5'-GGC TTT CTT GCA TTC CAC CA-3'	
SOX2	Forward	5'-GCTGGGCGCCGAGTGGA-3'	440bp
	Reverse	5'-GGCGAGCGTTCATGTAGGTCTG-3'	
HES 1	Forward	5'-AAA ATT CCT CCT CCC CGG TG-3'	119bp
	Reverse	5'-CCT TCG CCT CTT CTC CAT GA-3'	
GAPDH	Forward	5'-AGT TGT CAT GGA TGA CCT TGG C-3'	283bp
	Reverse	5'-ATC ACC ATC TTC CAG GAG CGA-3'	

Supplementary Table 3: Antibodies used for Western blot analysis.

Antibody	Molecular weight (kDa)	Clone No.	Dilution	Secondary antibody	Manufacturer	Catalogue No.
KITENIN	72KDa		1:2500	rabbit	Atlas	HPA025235
N-cadherin	100 KDa		5ug/ml (2mg/ml stock)	rabbit	Abcam	ab12221
ZEB1	125 Kda		1:500	rabbit	Bethyl	IHC-00419
ZEB2	157 Kda		1:500	rabbit	Sigma	HPA003456
SNAI1	29 KDa	H-130	1:1000	rabbit	Santa cruz	sc-28199
SLUG	30 KDa		1:500	rabbit	Abcam	ab38551
TWIST	21 KDa	Twist2C1a	1:1000	mouse	Abcam	ab50887
Actin	42 KDa	C4/actin	1:10000	mouse	BD transduction laboratories	612656

Supplementary Table 4: Univariate and multivariate analysis of predictors of overall survival in patients with glioma.

Variables		No.	Mean (weeks)	P-value (univariate)	P-value (multivariate)	Hazard ratio
Age	< 60 years	60	54.3	<0.001	0.001	1
	≥ 60 years	26	23.9			3.300
Sex	Male	41	44.4	0.797	0.465	1
	Female	45	44.5			0.762
Tumor size	< 4.5cm	46	46.4	0.165	0.335	1
	≥ 4.5cm	40	40.6			1.478
Location	Non-eloquent	43	40.2	0.179	0.754	1
	Eloquent	43	48.2			1.128
Edema	None to mild	40	43.4	0.870	0.284	1
	Moderate to severe	46	44.8			0.671
Cystic change	Absent	37	44.1	0.937	0.958	1
	Present	49	44.7			1.020
WHO grade	Low-grade (grade 1 or 2)	33	62.5	<0.001	0.005	1
	High-grade (grade 3 or 4)	53	33.5			4.251
Kitenin expression	Low	51	47.5	0.274	0.933	1
	High	35	32.8			0.933

Supplementary Table 5: Univariate and multivariate analysis of predictors of progression-free survival in patients with glioma.

Variables		No.	Mean (weeks)	P-value (univariate)	P-value (multivariate)	Hazard ratio
Age	< 60 years	60	45.9	<0.001	<0.001	1
	≥ 60 years	26	14.8			2.994
Sex	Male	41	33.9	0.443	0.330	1
	Female	45	37.9			0.723
Tumor size	< 4.5cm	46	39.7	0.076	0.412	1
	≥ 4.5cm	40	30.5			1.344
Location	Non-eloquent	43	29.9	0.031	0.473	1
	Eloquent	43	42.0			0.785
Edema	None to mild	40	37.4	0.530	0.759	1
	Moderate to severe	46	34.2			0.905
Cystic change	Absent	37	35.8	0.883	0.832	1
	Present	49	36.7			0.928
WHO grade	Low-grade (grade 1 or 2)	33	60.5	<0.001	<0.001	1
	High-grade (grade 3 or 4)	53	22.2			5.524
Kitenin expression	Low	51	42.0	0.041	0.499	1
	High	35	23.8			1.233

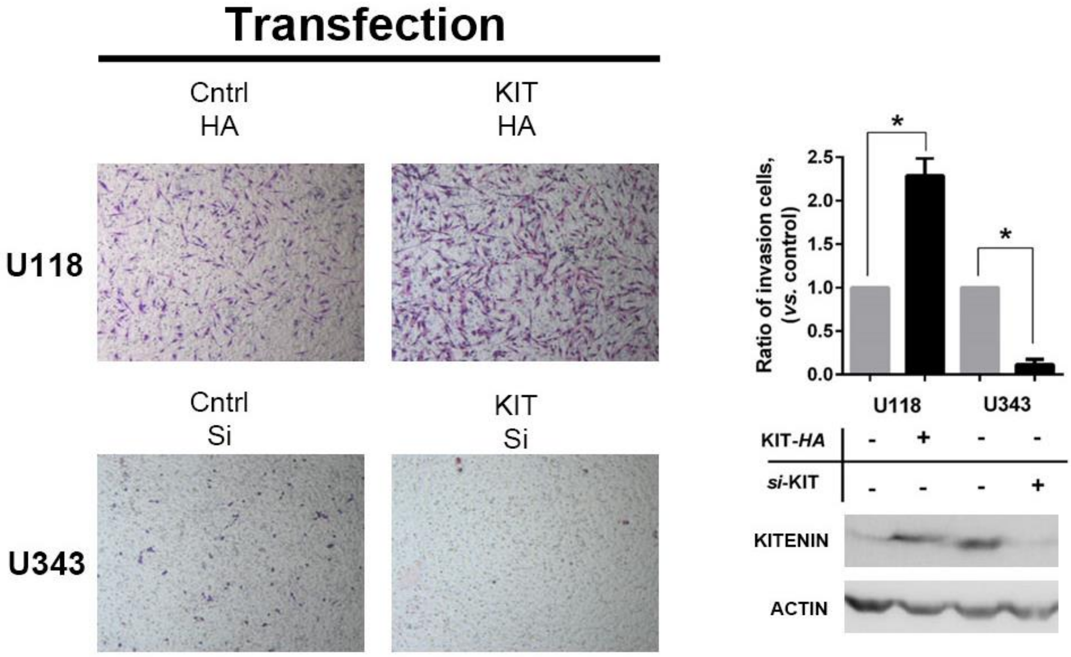

Supplementary Figure 1: Effect of KITENIN overexpression and knockdown on the invasive capacity of human glioma cell lines. A significantly larger number of KITENIN-overexpressing than mock-transfected U118 cells (mixed polyclonal, not single clone cells) migrated through the membrane. Transient knockdown of KITENIN in U343 cells decreased cell invasion. Results are presented as the relative ratio of invasive cells in the test compared with the control group for each cell line. Bar graphs show the mean ± standard error of mean (SEM). (*P<0.05).

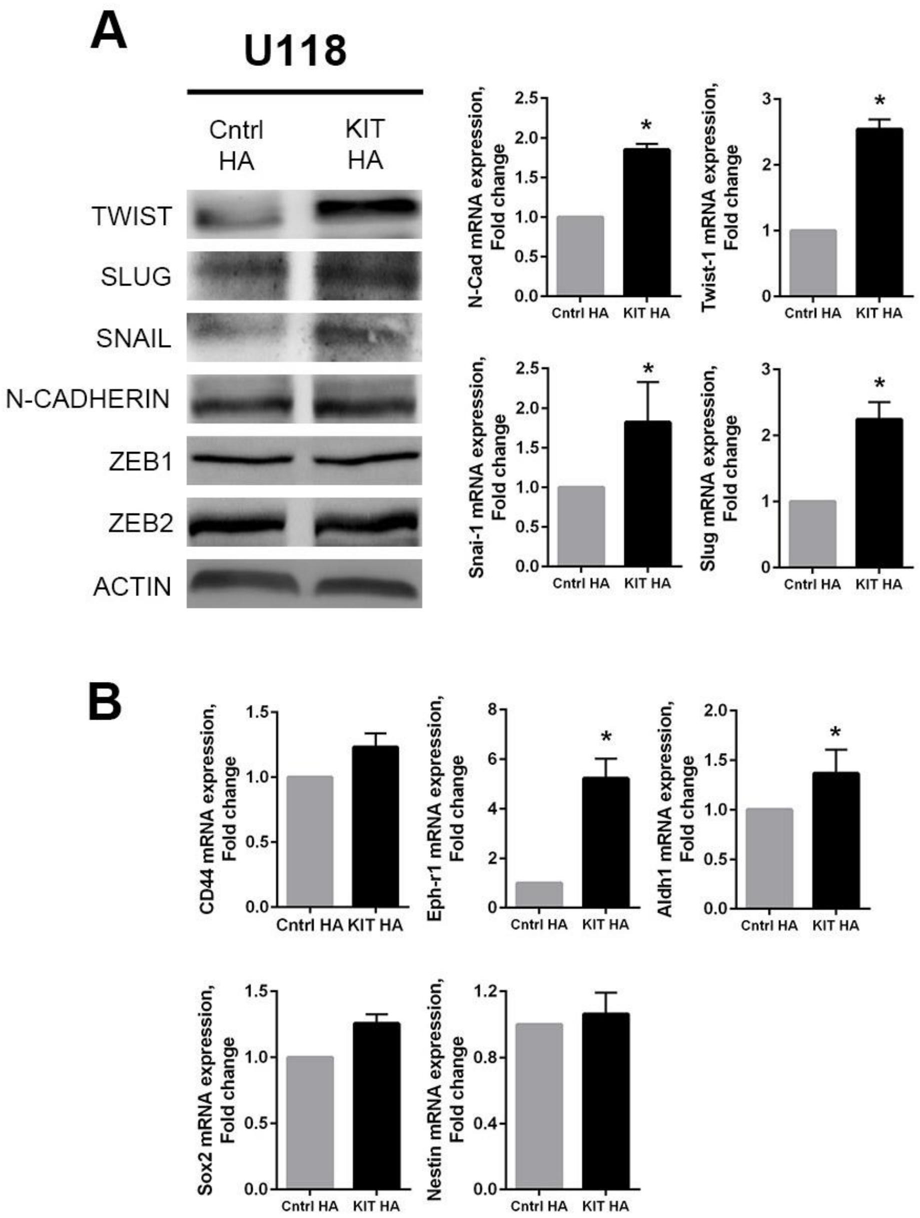

Supplementary Figure 2: EMT (A) and cancer stemness (B) markers in KITENIN-overexpressing U118 cells. KITENIN-overexpressing U118 cells (mixed polyclonal cells) showed increased expression of Twist, SNAIL, SLUG and N-cadherin protein, as shown by Western blotting, and mRNA, as shown by qRT-PCR, compared with mock-transfected U118 cells. Some markers related to cancer stemness, including EPH-B1, ALDH1, CD44, Sox2, and Nestin, were also increased. Compared with the results in GL261 overexpressing cells, the KITENIN-associated differences in expression of these markers were not as prominent as in GL261 cells, possible due to mixed clonality in KITENIN expression. Bar graphs show the mean ± SEM. (*P<0.05).

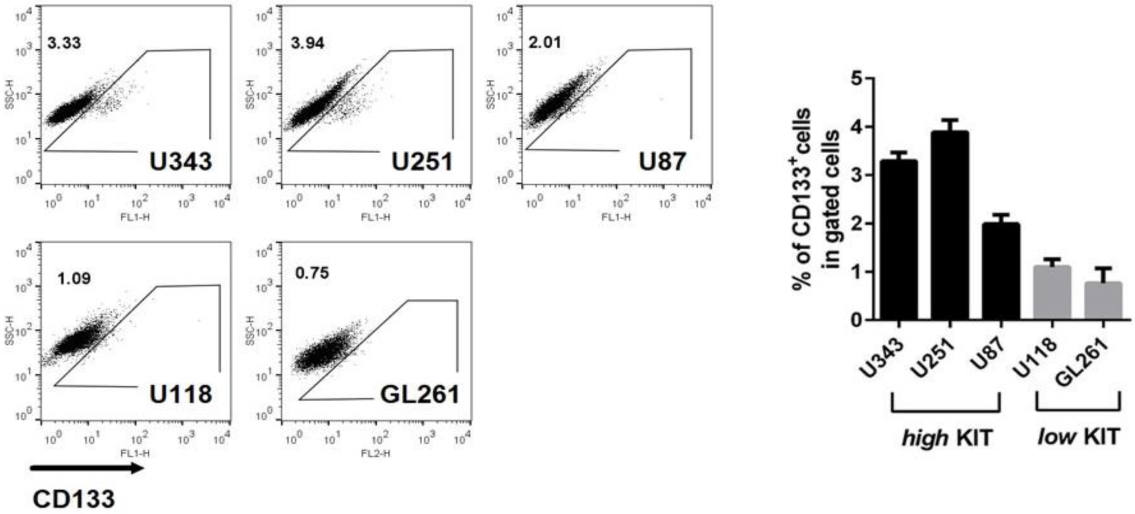

Supplementary Figure 3: Flow cytometry analysis of CD133 according to the endogenous KITENIN level in glioma cell lines. The proportion of CD133-positive cells was higher in glioma cell lines with high (U343, U251, and U87) than low (U118 and GL261) endogenous KITENIN expression.

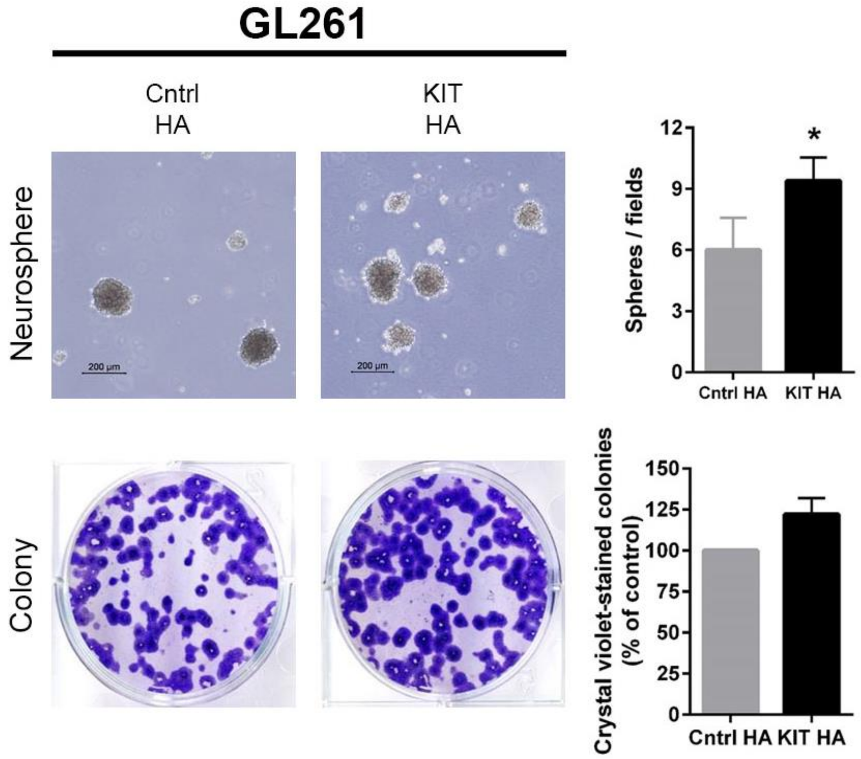

Supplementary Figure 4: Colony- and neurosphere-forming abilities of KITENIN-overexpressing GL261 cells. KITENIN overexpression increased the numbers of colonies and neurospheres. Bar graphs show the mean ± SEM. (*P<0.05).

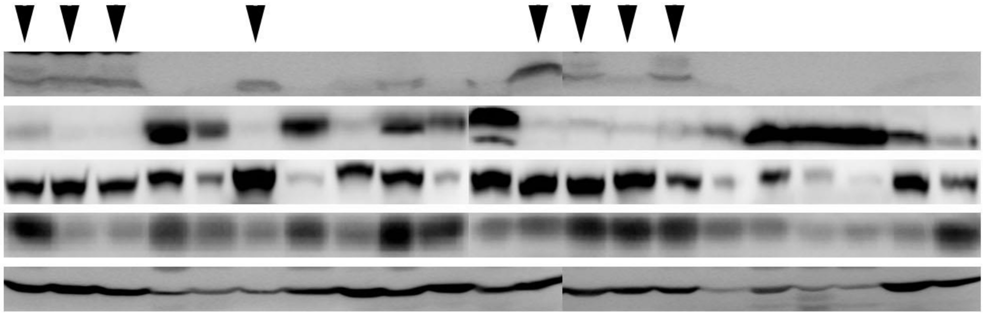

Supplementary Figure 5: Possible link of KITENIN with EMT markers in human glioma samples. Western blotting analysis showing that samples with high KITENIN expression (arrow) showed high expression of the mesenchymal marker N-cadherin and low expression of the epithelial marker E-cadherin. By contrast, samples with low expression of KITENIN showed high expression of E-cadherin and low expression of N-cadherin.
